# Computational design and characterization of a temperature-sensitive plasmid replicon for gram positive thermophiles

**DOI:** 10.1186/1754-1611-6-5

**Published:** 2012-05-11

**Authors:** Daniel G Olson, Lee R Lynd

**Affiliations:** 1 Thayer School of Engineering at Dartmouth College, Hanover, NH 03755, USA; 2 Department of Biological Sciences at Dartmouth College, Hanover, NH 03755, USA; 3 BioEnergy Science Center, Oak Ridge, TN, 38738, USA

**Keywords:** Temperature sensitive, Rolling circle, Plasmid, *Clostridium thermocellum*, Gram positive, Thermophile

## Abstract

****Background**:**

Temperature-sensitive (Ts) plasmids are useful tools for genetic engineering, but there are currently none compatible with the gram positive, thermophilic, obligate anaerobe, *Clostridium thermocellum*. Traditional mutagenesis techniques yield Ts mutants at a low frequency, and therefore requires the development of high-throughput screening protocols, which are also not available for this organism. Recently there has been progress in the development of computer algorithms which can predict Ts mutations. Most plasmids currently used for genetic modification of *C. thermocellum* are based on the replicon of plasmid pNW33N, which replicates using the RepB replication protein. To address this problem, we set out to create a Ts plasmid by mutating the gene coding for the RepB replication protein using an algorithm designed by Varadarajan et al. (1996) for predicting Ts mutants based on the amino-acid sequence of the protein.

****Results**:**

A library of 34 mutant plasmids was designed, synthesized and screened, resulting in 6 mutants which exhibited a Ts phenotype. Of these 6, the one with the most temperature-sensitive phenotype (M166A) was compared with the original plasmid. It exhibited lower stability at 48°C and was completely unable to replicate at 55°C.

****Conclusions**:**

The plasmid described in this work could be useful in future efforts to genetically engineer *C. thermocellum*, and the method used to generate this plasmid may be useful for others trying to make Ts plasmids.

## **Introduction**

Thermophilic bacteria such as *Clostridium thermocellum* show many promising features for industrial biotechnology and biofuel production [1,2]. For many years, a lack of genetic tools impeded progress in engineering these organisms [2]. Recently, however, several key tools relevant to engineering *C. thermocellum* have become available, including two antibiotic resistance markers, the *pyrF* uracil auxotrophic marker that can be used for both positive and negative selection [3,4], and the *hpt* and *tdk* markers that can be used for negative selection [5]. Positive and negative selection mechanisms can be combined to create a maker recycle system, which allows a theoretically unlimited number of cumulative mutations [5,6]. The use of auxotrophic markers, however, is strain-specific, and the benefit of Ts plasmids is that they have the potential to work in a wider range of strains.

Although Ts plasmids have been developed for many organisms [7-12], there are very few that are compatible with the 45°C-62°C growth range of *C. thermocellum*[13]. The traditional method of creating temperature-sensitive plasmids is to randomly mutate plasmid DNA (usually with hydroxylamine or error-prone PCR), transform the resulting library into the target organism and replica plate at different temperatures to screen for mutated plasmids that form colonies at a low temperature that permits replication, but not at a higher temperature where replication fails [7-9,14]. To ensure a high probability of finding the desired mutant, high transformation efficiency and a replica plating protocol are necessary to allow the screening of thousands of colonies. Neither of these techniques, however, is well developed for *C. thermocellum*.

**Figure 1:**
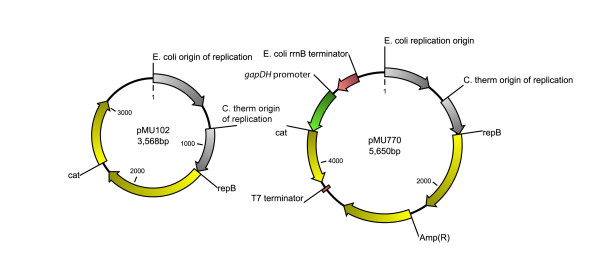
**Diagram of plasmids pMU102 and pMU770.** Yellow arrows indicate coding sequences, green arrows indicate promoters, red arrows indicate terminators, and grey arrows indicate replication origins. The *E. coli* origin of replication is derived from plasmid pUC19. The *C. thermocellum* origin of replication, *repB* replication gene, and *cat* antibiotic resistance gene are derived from plasmid pNW33N. The *gapDH* promoter is derived from *C. thermocellum* genomic DNA. The *rrnB* and T7 terminators were synthesized based on publicly available sequence data.

Currently plasmids pMU102 and pMU770 (Figure 1) are being used for genetic engineering of *C. thermocellum*[3,4]. These plasmids are based off of the *Bacillus* shuttle vector pNW33N [15], and are believed to replicate by the rolling circle method [16]. A key feature of rolling-circle plasmids is the replication protein, Rep. The *repB* gene from pNW33N (and pMU102 and pMU770) is identical to that of pUB110, a plasmid first isolated from thermophilic bacilli [17], which has been studied extensively as a model system for this type of plasmid [18].

Recent advances in understanding of protein folding have suggested that it may be possible to predict mutations that could cause a protein to be unstable at elevated temperatures. Hydrophilic amino acids and hydrophobic amino acids tend to associate with themselves. Since proteins are typically found in an aqueous environment, the hydrophobic amino acids are found buried in the core of the protein and play an important role in protein stability. It is possible to predict these buried, hydrophobic, amino acid residues solely from the amino acid sequence [19]. This technique has been successfully used to make Ts proteins in a variety of organisms [20]. We applied this technique to alter the stability of the RepB protein encoded by plasmids pMU102 and pMU770. The objective of this research was to alter the thermostability of a plasmid that functions in the 45°C-62°C growth range of *C. thermocellum*.

## **Results and discussion**

### **Initial screen**

**Figure 2:**
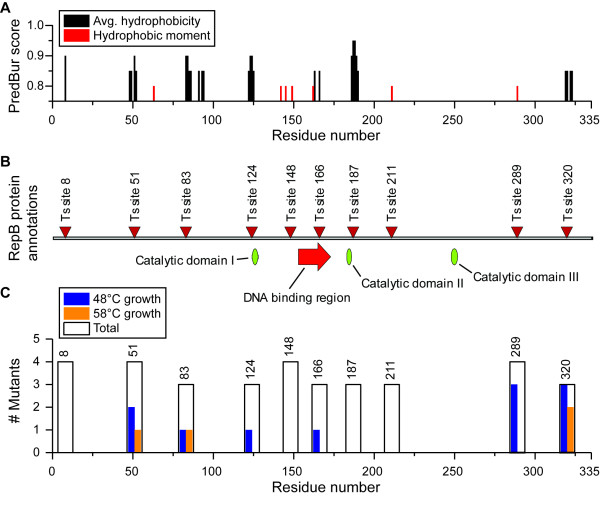
***RepB* sites chosen for mutagenesis.** Panel **A** represents the output of the PredBur program used to select sites for mutagenesis. Sites were chosen by the PredBur average hydrophobicity algorithm (black) or the PredBur hydrophobic moment algorithm (red). The PredBur score indicates the probability of burial of the residue. Higher PredBur values suggest a higher likelihood of the given residue being buried and thus a higher likelihood that mutation of that residue will result in a Ts plasmid. Panel **B** shows the 10 sites that were selected for mutagenesis (triangles) as well as annotation of putative catalytic domains (green ovals) [27] and a putative DNA binding region (red arrow) [28]. Panel **C** shows the results of the initial screen for temperature sensitivity for mutations in the pMU770 backbone. A black outline indicates the number of mutants generated at each site (3 or 4). Blue bars indicate the number of mutants able to confer thiamphenicol resistance at 48°C and orange bars indicate the number of mutants able to confer thiamphenicol resistance at 58°C. The difference between the blue bars and the orange bars indicates the number of temperature-sensitive mutants at each site.

A 34-member library of plasmids with mutated *repB* genes was designed, synthesized in the pMU770 plasmid backbone, and each member was individually transformed into *C. thermocellum*. Transformations were allowed to recover in liquid culture at 48°C in the absence of selection, and then each resulting strain was inoculated into liquid culture at both 48°C and 58°C in the presence of thiamphenicol to screen for differences in growth. The growth results are shown in Table 1 and Figure 2C. Growth at 48°C, but not at 58°C was considered an indication of temperature-sensitivity and the 6 mutants with this phenotype, I51E, M166A, I289W, I289L, I289Y and I320T, were chosen for further analysis.

**Table 1:** Initial library of RepB mutants

	**Amino acid residue**			**Growth**	
**Name**	**Position**	**Old**	**New**	**48°C**	**58°C**
770-M8C	8	Met	Cys	-	-
770-M8E	8	Met	Glu	-	-
770-M8G	8	Met	Gly	-	-
770-M8Y	8	Met	Tyr	-	-
770-I51E	51	Ile	Glu	+	-
770-I51W	51	Ile	Trp	-	-
770-I51L	51	Ile	Leu	+	+
770-I51C	51	Ile	Gln	-	-
770-V83A	83	Val	Ala	+	+
770-V83L	83	Val	Leu	-	-
770-V83R	83	Val	Arg	-	-
770-F124W	124	Phe	Trp	+/−	-
770-F124S	124	Phe	Ser	-	-
770-F124G	124	Phe	Gly	-	-
770-G148M	148	Gly	Met	-	-
770-G148P	148	Gly	Pro	-	-
770-G148T	148	Gly	Thr	-	-
770-G148Y	148	Gly	Tyr	-	-
770-M166A	166	Met	Ala	+	-
770-M166H	166	Met	His	-	-
770-M166G	166	Met	Gly	-	-
770-L187A	187	Leu	Ala	-	-
770-L187R	187	Leu	Arg	-	-
770-L187T	187	Leu	Thr	-	-
770-W211 D	211	Trp	Asp	-	-
770-W211T	211	Trp	Thr	-	-
770-W211V	211	Trp	Val	-	-
770-I289W	289	Ile	Trp	+	-
770-I289L	289	Ile	Leu	+	+/−
770-I289Y	289	Ile	Tyr	+	-
770-I289 D	289	Ile	Asp	-	-
770-I320T	320	Ile	Thr	+	-
770-I320F	320	Ile	Phe	+	+
770-I320Y	320	Ile	Tyr	+	+

Mutants of the *repB* gene were synthesized that contained a single codon substitution. Growth was determined by optical density measurements in a microplate reader. Measurements were performed in triplicate. ‘+’ indicates growth in all trials, ‘-‘ indicates no growth in all trials, ‘+/−‘ indicates growth in some (but not all) trials.

### **Change from pMU770 backbone to pMU102 backbone**

**Figure 3:**
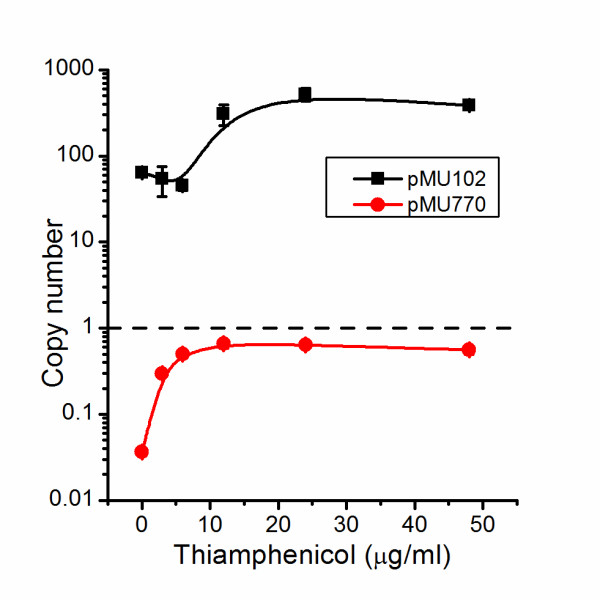
**Plasmid copy number for plasmids pMU102 and pMU770.** Copy number of cultures containing either plasmid pMU102 or pMU770 over a range of different thiamphenicol concentrations. Black squares represent plasmid pMU102. Red circles represent plasmid pMU770. In some cases error bars are obscured by the data point marker. Error bars represent one standard deviation, n = 3.

At this point we decided to reconstruct the 6 mutants exhibiting at Ts phenotype in the pMU770 plasmid backbone in the pMU102 plasmid backbone. The pMU770 backbone contains a 522 bp region from the *C. thermocellum glyceraldehyde 3-phosphate dehydrogenase (gapDH)* locus (which is used as a strong promoter for the *cat* gene). Unfortunately this region of homology is large enough to allow for unintended integration onto the chromosome [5], which could interfere with subsequent analysis. Measurement of the copy number of plasmids pMU102 and pMU770 showed that under most conditions pMU770 showed a copy number of ~1 (Figure 3). Since it is difficult for plasmids at this copy number to be stably maintained, it suggests that pMU770 may have been maintained by chromosomal integration instead.

Since the pMU102 plasmid backbone does not have any homology to the *C. thermocellum* chromosome, all 6 Ts mutants were reconstructed in this backbone and the resulting 6 plasmids (102-I51E, 102-M166A, 102-I289W, 102-I289L, 102-I289Y and 102-I320T) were individually transformed into *C. thermocellum*.

### **Characterization of growth kinetics and plasmid copy number**

**Figure 4:**
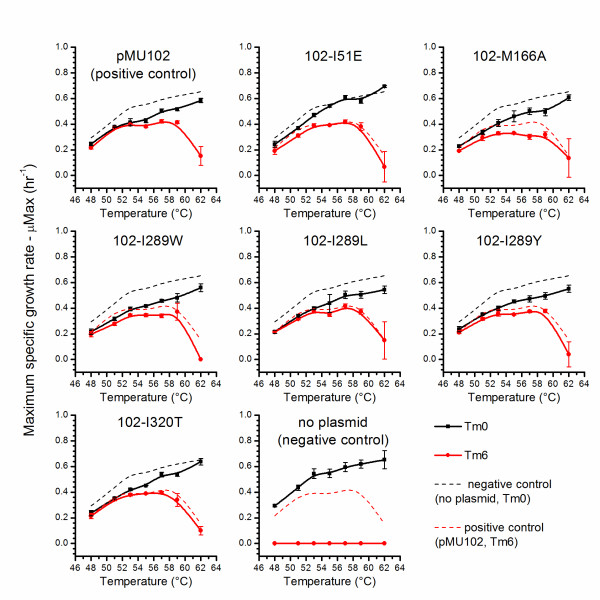
**Growth kinetics of plasmid-containing strains**. Maximum specific growth rate (μMax) was measured in the presence and absence of thiamphenicol at a range of temperatures for 6 strains of *C. thermocellum* containing potential temperature-sensitive plasmids and 2 control strains (plasmid pMU102 and no-plasmid). A solid black line indicates μMax in the absence of thiamphenicol. A solid red line indicates μMax in the presence of thiamphenicol. The dashed black line represents μMax of the no-plasmid control in the absence of thiamphenicol. The dashed red line represents the μMax of the positive control plasmid pMU102 in the presence of thiamphenicol. Error bars represent a standard deviation, n = 4.

**Figure 5:**
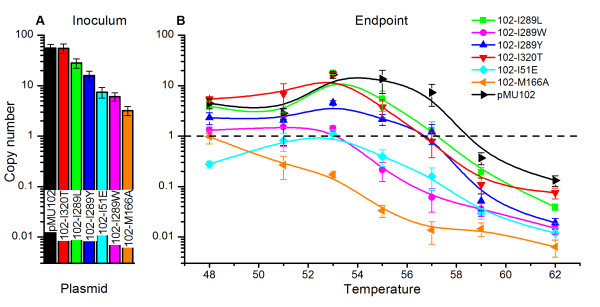
**Plasmid copy number vs. temperature.** Panel A shows the plasmid copy number in the inoculum. Panel B shows the endpoint copy number after ~7 generations of growth at a range of temperatures from 48°C to 62°C in media without antibiotic selection. Copy number was measured by qPCR. Error bars in both panels represent one standard deviation, n = 3.

The growth of plasmid-containing cultures was measured by changes in optical density at 600 nm (OD_600_). In the absence of selection, the wild type strain (no plasmid) had the highest specific growth rate, although the 102-I51E mutant exhibited a similar growth rate at temperatures above 55°C. In the presence of selection, cultures containing the 102-M166A and 102-I289W mutant plasmids had lower specific growth rates than those containing plasmid pMU102 (Figure 4). The plasmid copy number (PCN) of cultures grown without selection was measured by qPCR. Several of the plasmids showed lower copy number at elevated temperatures compared with the pMU102 control plasmid (Figure 5). Of all the plasmids tested in the absence of selective pressure, unmodified pMU102 was the most stable, maintaining a PCN > 1 at temperatures up to 59°C while 102-M166A was the least stable, maintaining a PCN >1 only at 48°C (Figure 5).

Growth rate in the presence of antibiotic selection can be used to determine the metabolic burden of plasmid maintenance. The plasmid exhibiting the highest metabolic burden was plasmid 102-M166A, which exhibited a μMax of 0.30 ± 0.02 compared with 0.42 ± 0.01 for pMU102 when both were grown at 57°C, a reduction of 29% (Figure 4). The next-largest metabolic burden was plasmid 102-I289W. Interestingly, both of these plasmids also exhibited lower copy number at a given temperature (Figure 5).

Growth rate (μ) in the absence of antibiotic selection can be used to determine the rate at which a plasmid is cured by comparing the growth rate of the plasmid-containing strain with the plasmid-free strain. Most plasmids exhibited plasmid curing behavior similar to plasmid pMU102 with the notable exception of 102-I51E (Figure 4). At some temperatures, the strain containing plasmid 102-I51E grew almost as fast as the no-plasmid control, suggesting that the plasmid was cured much more rapidly than pMU102. This plasmid also exhibited particularly low copy number at a given temperature (Figure 5). The difference in growth kinetics of the strain containing this plasmid suggests that it may have achieved temperature-sensitivity by a mechanism different from the other strains tested, and will be an interesting candidate for future mechanistic studies.

### **Plasmid curing**

**Figure 6:**
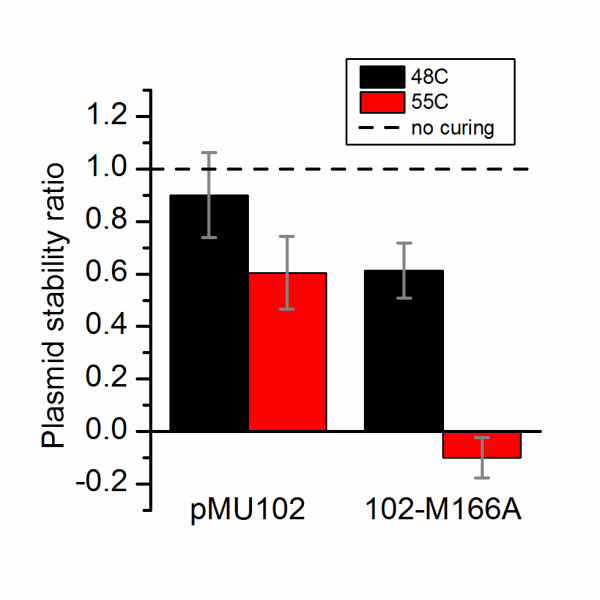
**Rate of plasmid curing.** Colony forming units (CFUs) were measured with and without thiamphenicol both at the beginning and end of growth in the absence of selection at 48°C and 55°C. These values were used to calculate the plasmid stability ratio (described in the text). A plasmid stability ratio of 1 implies stable replication. A plasmid stability ratio of 0 implies no replication. Error bars represent one standard deviation, n = 3.

To directly measure plasmid curing, strains containing plasmids pMU102 and 102-M166A were grown overnight in the absence of selection at either 48°C or 55°C and plasmid curing was measured by colony forming unit (CFU) counts on selective and non-selective media (Figure 6).

The CFU counts were transformed using the following formula for plasmid stability ratio (PSR)

(1) $$PSR=\frac{log_{10}(CFU_{antibiotic,\;final\;timepoint})-log_{10}(CFU_{antibiotic,\;initial\;timepoint})}{log_{10}(CFU_{no\;antibiotic,\;final\;timepoint})-log_{10}(CFU_{no\;antibiotic,\;initial\;timepoint})}$$

Where PSR = 1 indicates that the plasmid replicates stably, 0 < PSR < 1 indicates that the plasmid does not replicate stably, PSR = 0 indicates that the plasmid does not replicate at all (however any existing copies of the plasmid are maintained at their initial level in the population), and PSR < 0 indicates that the plasmid does not replicate at all (and existing copies of the plasmid are removed from the population, possibly by cell death). Plasmid pMU102 is relatively stable (PSR = 0.9 ±0.16) at 48°C, although the stability decreases at 55°C (PSR = 0.6 ±0.14). Plasmid 102-M166A is somewhat unstable at 48°C (PSR = 0.6 ±0.11) and completely unable to replicate at 55°C (PSR = −0.1 ±0.08). Comparing the PSR of plasmids pMU102 and 102-M166A at 48°C shows that the M166A mutation caused a general decrease in plasmid stability of about 1/3. If this had been the only reason for plasmid instability, we would have expected to see a PSR for 102-M166A of about 0.4. Instead we see at PSR of about 0, which suggests that this additional instability is specifically temperature-dependent.

### **Effect of amino acid substitutions**

Chakshusmathi et al. suggest two approaches for generating a temperature-sensitive protein [20]:

1. Introduce Asp (poorly tolerated at buried positions, because it is small, rigid, and charged) at two predicted buried sites. If the resulting mutants are inactive, then examine the remaining 18 mutants at each position for Ts behavior.

2. Introduce positively charged, polar, small and large hydrophobic residues (Lys, Ser, Ala, and Trp) at four predicted buried sites.

In this work, approach 2 was followed, but modified to include all amino acids (instead of just Lys, Ser, Ala, and Trp) and ten predicted buried sites were chosen instead of four. These modifications were chosen to maximize library diversity while minimizing cost. In the 34-member library, two mutants had Asp substitutions: W211 D and I289 D. In both cases the mutant protein was inactivated at both 48°C and 58°C. At site 211, two other substitutions were tested: Thr and Val. Both of these substitutions also resulted in inactive proteins, although Thr and Val are very different from Trp suggesting that substitution with a more-similar amino acid such as Tyr or Phe may allow for Ts behavior [21]. At site 289, all substitutions except for Asp resulted in a Ts mutant, which suggests that this site is conducive for the generation of Ts mutants. The PredBur algorithm identifies buried residues on the basis of average hydrophobicity and hydrophobic moment. Average hydrophobicity is used to identify buried residues that are not part of an ordered secondary structure, whereas hydrophobic moment is used to identify buried residues that *are* part of a secondary structure (i.e. one face of an α-helix or β-sheet). Site 289 was identified based on the hydrophobic moment calculation (Figure 2, panel A; Additional file 1: S1 text), suggesting that it may be part of a secondary structure that plays a key role in RepB structure. 22 of 34 mutants eliminated the ability of the plasmid to confer antibiotic resistance at either 48°C or 58°C (Figure 2), suggesting that the PredBur algorithm is able to identify destabilizing mutations with a high degree of reliability.

## **Conclusions**

In conclusion, a new Ts plasmid was developed for use *in C. thermocellum* using computational techniques that greatly reduced the size of the library that needed to be screened. Although random mutagenesis and screening techniques often require analysis of thousands of mutant plasmids [10], the mutant library generated with the PredBur program yielded 6 Ts plasmids out of 34 mutants, an improvement of over two orders of magnitude. Plasmid 102-M166A was the most temperature-sensitive, and exhibited a complete inability to replicate at 55°C. This plasmid adds yet another tool that should facilitate genetic engineering of this organism, and other gram-positive thermophiles. Furthermore, the strategy used to design this plasmid may prove useful for designing Ts plasmids for use with organisms that are distantly-related or have different growth temperatures.

## **Materials and methods**

### **Strains and media**

*C. thermocellum* strain DSM 1313 (WT) was grown in modified DSM 122 broth [3] with the addition of 50 mM 3-(N-morpholino) propanesulfonic acid (MOPS) sodium salt and 3 g/L trisodium citrate (Na3-C6H5O7·2 H2O). All manipulations were carried out inside an anaerobic chamber (Coy Laboratory Products Inc.) with an atmosphere of 85% nitrogen, 10% carbon dioxide, 5% hydrogen, and <5 ppm oxygen. Unless otherwise noted, cells were grown at 55°C using 5 g/L cellobiose as the primary carbon source. Transformation was performed by electroporation using a Bio-Rad Gene Pulser, using a 1.5 ms square pulse with a field strength of 13 kV/cm following the transformation protocol of Olson et al. [3].

### **Library design**

Our initial library of 34 mutants was designed as follows:

1. The PredBur algorithm [19] was used to predict sites in the amino acid sequence of RepB likely to contain buried amino acid residues.

2. Ten sites were chosen as targets for directed mutagenesis (Figure 2). Sites were selected based on high scores in the average hydrophobicity (>90% probability of being buried) or hydrophobic moment (>80% probability of being buried) calculations. In regions where several residues were predicted to be buried, only one site was chosen.

3. For each of these 10 sites, a SlonoMax SINGLE library (Sloning Biotechnology GmbH, Puchheim, Germany) was created comprising 3–4 mutants of the *repB* gene, from the pMU770 plasmid (Figure 2). Each mutant *repB* gene incorporated the most-frequent codon used by *C. thermocellum* for a given amino acid, although the choice of amino acid substitution was random.

The final library contained 34 members.

### **Specific growth rate measurements**

Freezer stocks were diluted 1:100 with growth media, then grown overnight with and without selection at a range of temperatures from 48°C to 62°C. Optical density measurements were performed in a Powerwave XS platereader [4] (BioTek Corporation, Winooski, VT) operated within the anaerobic chamber and specially modified by the manufacturer to allow incubation up to 68°C. OD_600_ readings were taken at 3-min intervals, then corrected for blank values and uniformly scaled to a 1 cm pathlength and log-transformed. Maximum specific growth rate (μMax) was determined by finding the maximum slope of log-transformed OD_600_ data using linear regression over a sliding 1 h window (20 data points). Data analysis was performed with the Gen5 software program (BioTek Corporation).

### **Plasmids**

Plasmids pMU102 and pMU770 were kindly supplied by Mascoma Corporation (Lebanon, NH). Plasmid pMU770 is derived from pMU102 with the following modifications: ampicillin resistance is provided by the *bla* gene from the pUC19 plasmid (Invitrogen Corporation), the *cat* gene from plasmid pMU102 was moved from its original location downstream of the *repB* gene and placed downstream of a 525 bp sequence thought to contain the *C. thermocellum* glyceraldehyde dehydrogenase (*gapDH*) promoter. This P*gapDH-cat* cassette is flanked upstream by the *E. coli rrnB* terminator and downstream by the T7 terminator to prevent unintended transcriptional readthrough (Figure 1).

### **Reconstruction of Ts mutations in pMU102 backbone**

The most promising candidates from the original 34-member library, consisting of 6 mutants, were reconstructed in the pMU102 backbone using the QuickChange II Site-Directed mutagenesis kit (Agilent Technologies, Inc., Santa Clara, CA). Plasmids were maintained in *Escherichia coli* TOP10 cells (Invitrogen Corporation, Carlsbad, CA) and prepared using QIAGEN Plasmid Mini kit (QIAGEN Inc., Valencia, CA). All 6 mutant plasmids were individually transformed into *C. thermocellum*. The resulting strains were grown at 51°C with 6 μg/ml thiamphenicol to make freezer stocks.

### **Plasmid copy number measurements**

Total DNA was prepared from 500–1000 μl of *C. thermocellum* cell culture grown to stationary phase using a ZR Fungal/Bacterial DNA Miniprep kit (Zymo Research Corporation, Irvine, CA). A Precellys 24 bead basher (Bertin Technologies, Montigny-le-Bretonneux, France) was used for the cell lysis step. An on-column digestion with the AvaI restriction enzyme (New England Biolabs, Ipswitch, MA) was performed to eliminate erroneous PCN measurements due to supercoiling [22] and to avoid size-bias during DNA extraction. After extraction, the resulting purified DNA was stored at −20°C for further analysis.

Plasmid copy number was measured using quantitative PCR (qPCR) [23-25], by comparing the ratio of the *cat* gene, which exists in single copy on the plasmid to the *celS* gene, which exists in single copy on the *C. thermocellum* chromosome [3]. All samples were measured in at least triplicate. Quantitative PCR was performed using iQ SYBR Green Supermix (Bio-Rad Corporation) in a CFX96 qPCR machine (Bio-Rad Corporation) with an annealing temperature of 50°C and other cycling parameters as suggested by the iQ SYBR Green Supermix datasheet. Data analysis was performed using Bio-Rad CFX manager software, version 2.1. For each sample, Cq was determined using the regression method. Copy number was determined using the ΔΔCq method with *celS* as the reference gene and automatic correction for primer amplification efficiency [26]. Primers XO7706 (5’-TCTCTGGTATTTGGACTCC-3’) and XO7707 (5’- CAGGTATAGGTGTTTTGGG-3’) were used to amplify a 117 bp region of the *cat* gene. Primers XO7712 (5’- CTCATCCGTCAATAGAAGAG-3’) and XO7713 (5’- TAAACAGCCTGTATAGCACG-3’) were used to amplify a 139 bp region of the *celS* gene. Primers for the *celS* gene were searched against the *C. thermocellum* chromosome for uniqueness using the BLAST algorithm. For XO7712, the next-highest BLAST hit had an expectation value of 371 and for XO7713, the next-highest BLAST hit had an expectation value of 24. Additional confirmation of the uniqueness of primers was that melt curves showed a unique melting temperature of 76.0°C for the *cat* gene amplicon and 81.5°C for the *celS* gene amplicon. During the optimization of the PCR protocol, the resulting PCR products were run on a 2% agarose gel and a single band was observed for each amplicon (data not shown). A control plasmid, pDGO-28, was synthesized with a single copy of the *cat* qPCR target and a single copy of the *celS* target using the miniGENE service from IDT (Integrated DNA Technologies, Inc., Coralville, IA) [24]. Primer amplification efficiency was measured using 9 10-fold serial dilutions of AvaI-linearized pDGO-28 plasmid. The amplification efficiency of the XO7706-XO7707 primer pair (*cat* gene) was 100.8%. The amplification efficiency of the XO7712-XO7713 primer pair (*celS* gene) was 97.1%. The average Cq of the *celS* gene was 19.12, and the average Cq of the *cat* gene no template control (NTC) was 31.09, resulting in a limit of detection of 2.5x10^-4^ for measuring plasmid copy number.

### **Plasmid curing measurement**

To measure plasmid curing, plasmid-containing cultures were grown at 48°C in the presence of 6 μg/ml thiamphenicol to an OD_600_ > 0.5 and plated on both selective (3 μg/ml thiamphenicol) and non-selective media and incubated at 48°C to measure the number of CFUs initially present. Then they were diluted 1:1000 into fresh media without selection and incubated at either 48°C or 55°C until OD_600_ > 0.5 and then plated again on both selective (3 μg/ml thiamphenicol) and non-selective media and incubated at 48°C to measure the change in CFUs.

## **Competing interests**

LRL is a founder of the Mascoma Corporation, which has a financial interest in genetic engineering of *Clostridium thermocellum*. DGO is a former employee of the Mascoma Corporation.

## **Authors’ contributions**

DGO designed the study, performed the experiments and analyzed the data. DGO and LRL wrote the manuscript. All authors read and approved the final manuscript.

## Supplementary Material

Additional file 1 **S1 text. Output from the PredBur algorithm.** Text output of the PredBur program showing the sites in the amino acid sequence of the RepB replication protein most likely to contain buried amino acid residues. Click here for file
