# Supplementary material for: Computational design and characterization of a temperature-sensitive plasmid replicon for gram positive thermophiles
Source: J Biol Eng. 2012 May 11;6:5. doi: 10.1186/1754-1611-6-5 (PMC3464808; doi:10.1186/1754-1611-6-5)
Supplement: Additional file 1 — S1 text. Output from the PredBur algorithm. Text output of the PredBur program showing the sites in the amino acid sequence of the RepB replication protein most likely to contain buried amino acid residues. [file 1754-1611-6-5-S1.pdf]

```
 ************************************************************
     Results with the Sequence in the data file  repB.seq
 ************************************************************
-----------------------------------------------------------------
```

P R E D I C T I O N/S F O R 95% B U R I A L:
~ ~ ~ ~ ~ ~ ~ ~ ~ ~ ~~~ ~ ~ ~ ~~~ ~ ~ ~ ~ ~ ~ ~
(BASED ON AV. HYDROPHOBICITY VALUES)

==

From Prediction

Res. No.	Res. buried	Av. Hydrophobicity	Window
187	LEU	85.350	MHV [L] VCV
188	VAL	76.920	HVL [V] CVE

Total number of residues in the data set =   334
Number of residues identified as buried =   2 (.599 %)

P R E D I C T I O N/S F O R 90% B U R I A L:
~ ~ ~ ~ ~ ~ ~ ~ ~ ~ ~~~ ~ ~ ~ ~~~ ~ ~ ~ ~ ~ ~ ~
(BASED ON AV. HYDROPHOBICITY VALUES)

==

From Prediction

Res. No.	Res. buried	Av. Hydrophobicity	Window
8	MET	65.200	FNI [M] CPN
51	ILE	75.820	LLH [I] LEF
83	VAL	77.290	LYR [V] WFC
84	TRP	65.200	YRV [W] FCK
123	LEU	72.890	VRW [L] FLT
124	PHE	72.530	RWL [F] LTL
186	VAL	82.420	HMH [V] LVC
187	LEU	85.350	MHV [L] VCV
188	VAL	76.920	HVL [V] CVE
189	CYS	71.790	VLV [C] VEP

Total number of residues in the data set =   334
Number of residues identified as buried =   10 (2.994 %)

P R E D I C T I O N/S F O R 80% B U R I A L:
~ ~ ~ ~ ~ ~ ~ ~ ~ ~ ~~~ ~ ~ ~ ~~~ ~ ~ ~ ~ ~ ~ ~
(BASED ON AV. HYDROPHOBICITY VALUES)

==

From Prediction

Res. No.	Res. buried	Av. Hydrophobicity	Window

8	MET	65.200	FNI [M] CPN
48	LEU	72.530	YFE [L] LHI
49	LEU	75.820	FEL [L] HIL
51	ILE	75.820	LLH [I] LEF
52	LEU	63.740	LHI [L] EFK
83	VAL	77.290	LYR [V] WFC
84	TRP	65.200	YRV [W] FCK
85	PHE	61.540	RVW [F] CKS
86	CYS	61.540	VWF [C] KSR
91	CYS	66.670	SRL [C] PMC
93	MET	73.260	LCP [M] CNW
94	CYS	65.570	CPM [C] NWR
122	TRP	72.890	TVR [W] LFL
123	LEU	72.890	VRW [L] FLT
124	PHE	72.530	RWL [F] LTL
125	LEU	74.730	WLF [L] TLT
163	VAL	61.170	KNL [V] GFM
166	MET	64.100	VGF [M] RAT
186	VAL	82.420	HMH [V] LVC
187	LEU	85.350	MHV [L] VCV
188	VAL	76.920	HVL [V] CVE
189	CYS	71.790	VLV [C] VEP
190	VAL	65.930	LVC [V] EPT
319	ILE	72.160	GFS [I] IAM
320	ILE	76.920	FSI [I] AMW
322	MET	74.730	IIA [M] WNW
323	TRP	65.200	IAM [W] NWE

Total number of residues in the data set = 334
Number of residues identified as buried = 27 (8.084 %)

P R E D I C T I O N/S F O R 80% B U R I A L:
~ ~ ~ ~ ~ ~ ~ ~ ~ ~~~ ~ ~ ~ ~~~ ~ ~ ~ ~ ~ ~ ~

(BASED ON HYDROPHOBIC MOMENT VALUES)

===

From Prediction

Res. No.	Res. buried	Hydrophobic Moment	Window
63	CYS	245.390	RVKD [C] AEIL
142	LEU	225.520	LNKS [L] SDMA
145	MET	204.300	SLSD [M] AQGF
149	PHE	229.850	MAQG [F] RRMM
162	LEU	212.960	INKN [L] VGFM
211	TRP	253.590	WIQF [W] KKAM
289	ILE	272.780	LLKE [I] HKKL

Total number of residues in the data set = 334
Number of residues identified as buried = 7 (2.096 %)
